# The Effect of the In-Situ Heat Treatment on the Martensitic Transformation and Specific Properties of the Fe-Mn-Si-Cr Shape Memory Alloys Processed by HSHPT Severe Plastic Deformation

**DOI:** 10.3390/ma14164621

**Published:** 2021-08-17

**Authors:** Carmela Gurau, Gheorghe Gurau, Felicia Tolea, Bogdan Popescu, Mihaela Banu, Leandru-Gheorghe Bujoreanu

**Affiliations:** 1 Faculty of Engineering, “Dunarea de Jos” University of Galati, Domneasca Street 47, 800008 Galati, Romania; Carmela.Gurau@ugal.ro (C.G.); Gheorghe.Gurau@ugal.ro (G.G.); 2 National Institute of Materials Physics, Atomistilor 405A, 077125 Magurele, Romania; bogdan.popescu@infim.ro; 3 Department of Mechanical Engineering, University of Michigan, 2350 Hayward Street, Ann Arbor, MI 48109, USA; mbanu@umich.edu; 4 Faculty of Materials Science and Engineering, The “Gheorghe Asachi” Technical University of Iasi, Blvd. Dimitrie Mangeron 61A, 700050 Iasi, Romania; leandru-gheorghe.bujoreanu@academic.tuiasi.ro

**Keywords:** severe plastic deformation, HSHPT, ferromagnetic shape memory alloy, martensitic transformation, Fe-Mn-Si-Cr, magnetic properties, transport properties

## Abstract

This work focuses on the temperature evolution of the martensitic phase ε (hexagonal close packed) induced by the severe plastic deformation via High Speed High Pressure Torsion method in Fe_57_Mn_27_Si_11_Cr_5_ (at %) alloy. The iron rich alloy crystalline structure, magnetic and transport properties were investigated on samples subjected to room temperature High Speed High Pressure Torsion incorporating 1.86 degree of deformation and also hot-compression. Thermo-resistivity as well as thermomagnetic measurements indicate an antiferromagnetic behavior with the Néel temperature (T_N_) around 244 K, directly related to the austenitic γ-phase. The sudden increase of the resistivity on cooling below the Néel temperature can be explained by an increased phonon-electron interaction. In-situ magnetic and electric transport measurements up to 900 K are equivalent to thermal treatments and lead to the appearance of the bcc-ferrite-like type phase, to the detriment of the ε(hcp) martensite and the γ (fcc) austenite phases.

## 1. Introduction

The ability of some alloys to remember their shape at two different temperatures is called shape memory effect (SME), and the respective materials are referred to as shape memory alloys (SMAs). SME is governed by a non-diffusive first-order phase transformation called martensitic transformation (MT). During the MT the atoms move at distances smaller than the inter-atomic distances, and they go from an austenitic phase of high temperature and high symmetry (cubic type) to a martensitic phase of low temperature, with lower symmetry (tetragonal, orthorhombic, hexagonal). NiTi alloys, Cu-based and Fe-based alloys are the most well known types of SMAs. Based on their crystalline structure in the austenitic phase they are grouped in “β-type” with body centered cubic (bcc) cell structure, a subclass of them being Ni-Ti [1], Co-Ni-Ga [2,3,4], Cu-Zn-Al, Cu-Al-Ni [5] and Ni-Mn-Ga [6,7,8], Ni-Fe-Ga Heusler alloys [8,9] and “γ-type”, with face centered cubic (fcc) cell structure, comprising Fe-Mn and Fe-Mn-Si [10,11], FePd [12] or FeNiCoTi [13]. Among these, Fe-Mn shape memory alloys present a good workability, weldability, corrosion resistance [14], large damping effect [15], recovery strain due to the shape memory effect (SME) [16] and superelasticity [17,18]. It has been shown that addition of Si atoms enhances reversibility of martensite and the shape memory effect in the Fe-Mn alloys [16], while the Cr addition (between 5 and 9 wt.%) improves the corrosion resistance of Fe-Mn-Si alloys [19]. The MT of Fe-Mn-Si alloys shows a particular non-thermoelastic stress-induced γ‒ε martensitic transformation and its ε‒γ reverse transformation during subsequent heating [10,11,14,15,16,17,18,19,20,21]. Additionally, beside ε-martensite (hexagonal close packed—hcp—stress-induced structure), the α’-martensite (body-centred-tetragonal—bct—structure) may appear at the intersections of crystallographic defects induced by plastic deformation such as mechanical twins, stacking faults and shear bands [22,23]. In addition, according to [11], the α’-martensite can be formed in the stress-induced ε martensite, in a probable **γ-ε**-α’transformation, with the ε-martensite acting as an intermediate phase for α’-formation [23].

In the recent years, material properties design garnet high interest and consequently, the relationship between the process parameters and material properties requires more understanding. For example, it is not fully known which are the physical processes occurring during severe plastic deformation (SPD) of solid state materials which lead to significant changes of the typical properties of the raw material [24,25,26,27,28,29]. Thus, numerous methods of SPD have been developed to refine the microstructures of SMAs [30]. The possibility to control the grains size through SPD opens an opportunity to control the γ-ε martensitic transformation in shape memory ferrous alloys. Studies show that the ultrafine grained iron based SMAs exhibit excellent recoverable strain, being more beneficial in the long-term engineering applications [26,31]. Our previous work on Fe-28Mn-6Si-5Cr (wt.%) SMA also show that an (ultra)fine structure is obtained when the samples are subjected to SPD via high speed high pressure torsion (HSHPT) technique [32,33]. In contrast with most well known SMAs (NiTi and Cu based) to which the shape recovery is due to two-way martensitic transformation, hinge on ordered lattice structure, the Fe-Mn-Si SMAs are characterized by disordered lattice and a dislocation-assisted transformation mechanism [34].

The current work investigates on how the SPD in particular High Speed High Pressure Torsion (HSHPT) process influences the magnetic behavior of Fe_57_Mn_27_Si_11_Cr_4_ at % shape memory alloy during ultrafine microstructure development. The microstructure of the materials is investigated with emphases on the stacking faults, twinning and dislocation density in austenite, and correlated to the thermo-magnetic and transport properties. The results aim to extend the understanding of the application potential of SPD to the economically attractive iron based SMAs. Potential applications include pipe steel joints [35], fishplate for crane rail [35] or in constructions as dumping materials, where the stress induced ε martensite and deformation twins occur and disappear by cyclic tensile and compressive deformation which absorbs vibrations.

## 2. Material and Methods

**Figure 1:**
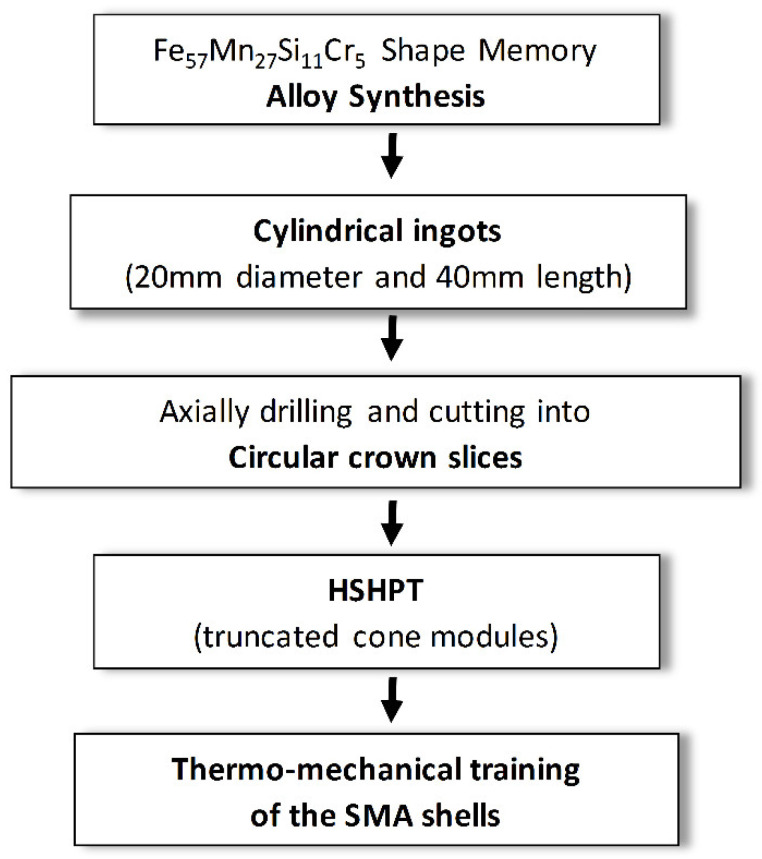
Schematic diagram of the experiment.

The Fe_57_Mn_27_Si_11_Cr_4_ at % SMA ingots were obtained by melting the elements in a Fives Celes induction furnace MP25 (Lautenbach, France). Before the SPD processing, circular crowns billets were cut out of the drilled ingots. In order to obtain ultra-fine grained samples via HSHPT process, three deformation parameters were adjusted: the rotation speed of the superior punch (~1.0 × 10^3^ rpm), the compression force at about ~1 GPa and the concurrent friction occurring between the punches and the sample (caused by the high pressure on the bottom anvil and to relative displacement of the sample-lower anvil). A lubricated billet was placed between the anvils. The specimens were initially pressed at 5 MPa at room temperature (RT). The resulted HSHPT processed specimens, under an applied pressure of about 1 GPa and 1.86 strain level (sample denoted C), are truncated cone-modules of ~30 mm in diameter, and 0.2 mm in thickness. The extent of deformation degree was estimated using the logarithmic relationship:

ε = ln (h_i_/h_f_) (1)

where h_i_ and h_f_ indicate initial and final thickness of the sample, respectively. The applied rotation speed of the upper piston was 1794 rpm. The duration of the process was around 10 s. After severe plastic deformation the conical SMA modules were pressed back into disk shapes. Further, thermo-mechanical cycles of simultaneous compression and heating at 450 °C were applied to improve the super-elastic properties of the modules (denoted TC). The processing pathway of the samples is presented in the schematic diagram of the experiment from Figure 1.

**Figure 2:**
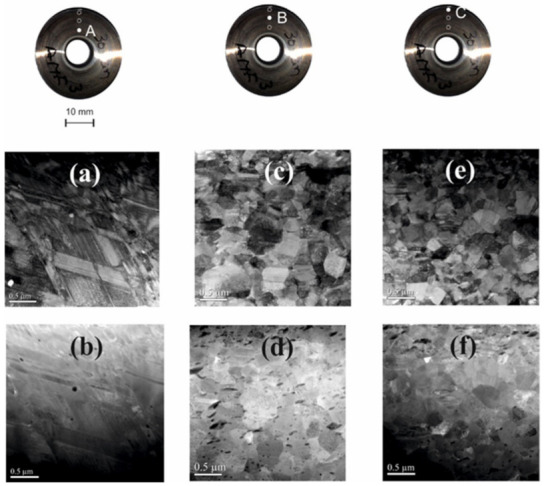
Truncated shells after HSHTP in three different radial areas, and the corresponding TEM and ADF micrographs grouped as follows: area (**A**) (**a**,**b**), area (**B**) (**c**,**d**) and area (**C**) (**e**,**f**).

All the subsequent investigations were carried out on samples taken from the peripheral area of the truncated shells (see Figure 2).

The chemical composition was performed using SPECTRO XEPOS 03 energy dispersive X-ray fluorescence (ED-XRF) spectrometer manufactured by SPECTRO Analytical Instruments GmbH, Kleve, Germany. The microstructure of the samples was studied by transmission electron microscopy (TEM) using a JEOL 2010F microscope (JEOL, Peabody, MA, USA) operated at 200 kV, with STEM mode. TEM specimens were ion milled using a Helios 650S instrument (Thermo Fisher Scientific, Waltham, MA, USA). Observations were made in bright field imaging modes. The samples for TEM investigations were taken perpendicularly to the surface of severe plastic deformation of the modules. Annular dark-field (ADF) was employed to improve grain visibility. The average grain size was calculated using ADF images and averaging the intersect lengths over 500 grains in different areas of the images. The crystalline structure was investigated by X-ray diffraction at RT using a Bruker AXS D8 Advance diffractometer (Cu Kα radiation) manufactured by Bruker, Hamburg, Germany), within the representative region of 2θ between 40° and 85°, in the Bragg–Brentano geometry. The phase transformation temperatures were determinate by differential scanning calorimetry (DSC using Netzsch 204 F1 Calorimeter, Selb, Germany, with Proteus Software 2007) with a scanning rate of 20 K/min and 10 K/min. The low temperature magnetic measurements below 350 K were performed with a Quantum Design—Superconducting Quantum Interference Device (SQUID) magnetometer in Reciprocal Space Option (RSO mode) in the temperature range from 4 K up to 390 K, manufactured in San Diego, CA, USA. The high temperature magnetic properties were estimated using the oven module of the Quantum Design-Physical Properties Measurement System (PPMS)—in Vibrating Sample Magnetometry (VSM) mode, manufactured in San Diego, CA, USA. The temperature and field-dependent resistivity in low and high temperature range were analysed in order to characterize the electrical transport of samples. These investigations were carried out in standard four-probe method (silver paste was used to make the contacts) using transport option of PPMS. The measurements have been carried out with the current along the longitudinal direction of samples, the magnetic field perpendicular to the samples.

Noteworthy, the magnetic and electric transport measurements were performed up to 970 K (high temperature) in controlled atmosphere, equivalent to an in situ thermal treatment which influences the constituent crystalline phases of the samples. For this reason, these samples are denoted further HC (for sample C after high temperature investigations) and HTC (for high temperature investigated TC sample).

## 3. Results and Discussion

### 3.1. TEM Observations

SMA modules were analyzed to understand the influence of the HSHPT processing on microstructural features visible under TEM. TEM observations enable characterization of the grain size and morphology, and they are important data for explaining the shape memory alloy behavior post SPD processing. Figure 2 shows the TEM and ADF micrographs observed on the cone generator, from the three distinct radially distributed areas, denoted A, B and C, respectively.

**Figure 3:**
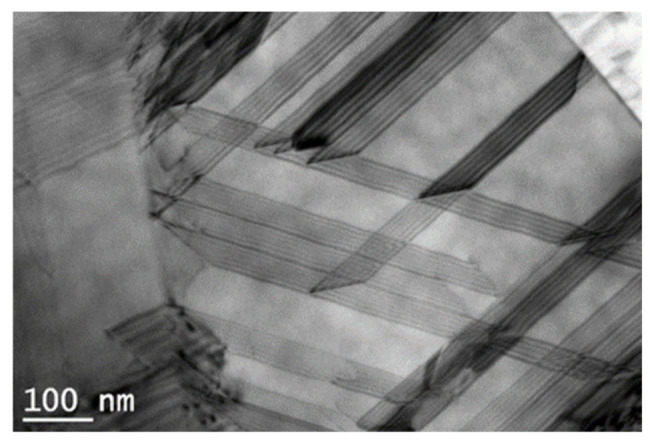
Stacking faults observed in the iron-based modules—processed by HSHPT (sample C).

The three analysed areas shown microstructural differences caused by the non-uniform severe plastic deformation of each region. Figure 2a shows TEM micrograph of the central part of the module where bands of martensite plates alongside stacking faults (Sfs), oriented mainly on two directions, can be identified. As it is already known, the fcc-to-hcp transformation process is promoted by gliding of multiple Shockley partials of 1/6 fcc type on alternate {111} fcc planes that depends on crystal orientation [34]. The stacking fault energy (SFE) of these alloys is lower than 20 mJ/m^2^ with consequences on the susceptibility to nano-sized twins in the microstructure. The gliding of Shockley partials that induce the γ-ε martensitic transformation depends on crystal orientation. Certain crystallographic textures in polycrystals will contribute to a better shape recovery at lower applied stresses [26]. It is well known that various factors are involved in the grain refinement of SPD—processed metals such as the intrinsic characteristics of material, the true strain, the temperature of deformation. In addition, it was reported that the grain size obtained in the SPD process of fcc materials is closely related to SFE value. A low value of γ SFE (below 20 mJ/m^2^), indicates a lower average grain size [36]. As a result of the HSHPT deformation, typical HCP stress-induced ε-martensite plates formed as parallel stacks of fine bands. In addition, a small amount of phase, α’ martensite, as little particles with tangle shape were distinguished (Figure 2a,b and the high magnification bright field TEM image from area A seen in Figure 3) at intersections in the ε-martensite due to the mutual dislocation reaction of different martensitic shear systems [37]. The formation of the α’martensite crystals has been established to be mainly associated with dislocations in the prior austenite phase and they appear in dislocated austenite areas [26,38,39].

In the B and C areas forming of martensite crystals among the austenite grains is noticed (as shown in Figure 3). The ε-martensite that appear inside the grain boundaries as fine parallel stripes is considered a prerequisite for the SME. In addition, frequently, the appearance of two or three martensite variants are reported within parent austenitic grains in the Si ≥ 4 wt.% alloys [37]. Plastic deformation of low SFEs Fe-Mn-Si based SMAs involves formation of stacking faults, mechanical twins, and shear bands [29]. In these B and C areas of the samples, ultra-fine austenitic grains are observed as a result of stronger compression and rotation during HSHTP process (Figure 2c–f). The average grain size in the B and C areas are determined by the linear-intercept method. In the middle (B) area of disk the average of austenitic grains is about 210 nm, while at the edge of the sample (C) the average grain size is under 190 nm. However, the values of the grain size in both areas are ranging between 10 nm and 500 nm. Within the ultra-fine γ grains the ε martensite stripes, under 50 nm wide, are visible. In the C region, the TEM micrograph shown in Figure 3, reveals extended parallel stacking faults with hcp structure type formed as a result of deformation by HSHPT.

### 3.2. The Mechanical Properties of Ultra-Fine Structured Fe-Mn-Si-Cr Alloy

**Figure 4:**
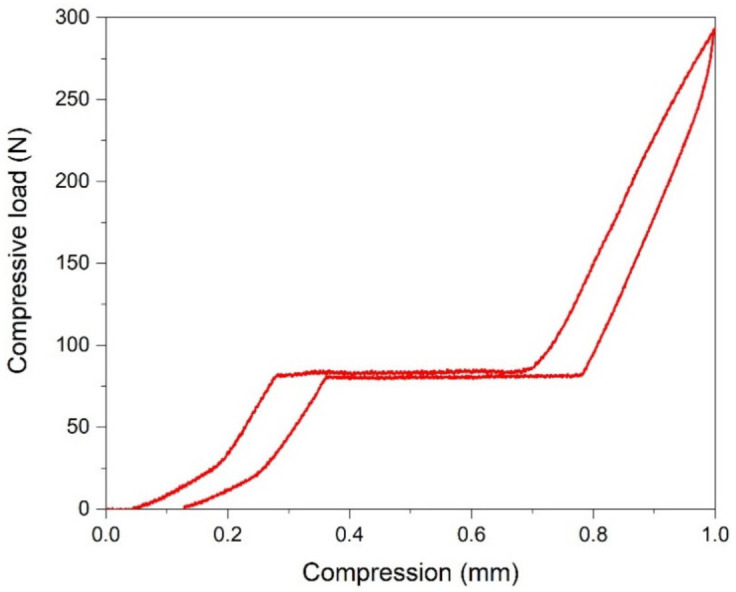
The shape memory response of Fe-Mn-Si-Cr SMA modules manufactured by HSHPT obtained during the compression loading/unloading cycles.

The elastic properties in Fe-Mn based alloy are related to a disordered distribution of magnetic moment [40]. The deformation induced by HSHTP promotes the refinement of the grain size, with an average under 200 nm, distributed on samples radius and contributes also to the enhancement of the SME. The curves obtained during the compression loading/unloading cycle of the analyzed truncated modules are shown in Figure 4. They suggest a “super-elastic” behavior showing a 92.85% recovery after deformation.

### 3.3. XRD Results

**Figure 5:**
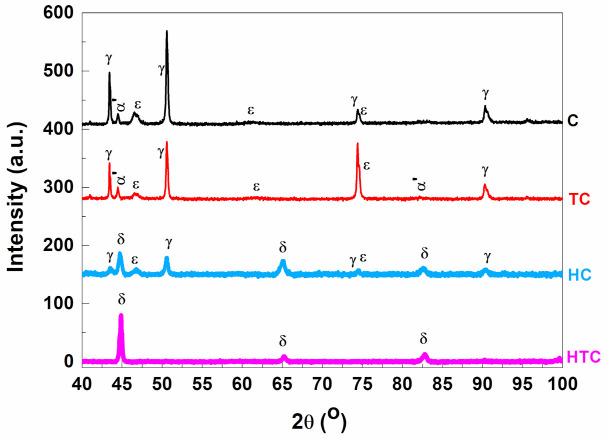
XRD patterns showing phase composition of C and TC alloys, and their evolution after the in situ heat treatment at 975 K.

The X-ray diffraction analysis was performed in the bulk state, on HSHPT’ed modules. Prior to XRD, etching was used to remove the oxidation of the surface layer and the regions affected by the cutting process. XRD investigations support the TEM observations showing that the SPD samples (C and TC) consist of the parent austenite γ phase (F*m-3m*, no. 225), the deformation induced ε martensite phase (P*63/mmc*, no. 194) and traces of α’ martensite (I*m-3m*, no. 229) (see Figure 5). However, after applying the in situ thermal treatment at 970 K the phase composition changes dramatically, with an δ-ferrite (bcc) [19] phase evolving toward becoming the dominant phase in both HC and HTC samples. Small amounts of the secondary phases, γ (fcc) and ε (hcp), are still detected in the HC sample, while through analysing the diffraction patterns of the HTC sample it can be counted a very weak contribution of the diffraction lines of the γ austenite. Our findings on the presence of second phases agree with the reports on FeMnSi-based systems, widely discussed in the literature [19]. A detailed structural analysis is beyond the scope of this study but also not possible due to the surface quality and the quantity of the samples resulted after the SPD, and moreover due to the samples hardness which prevented the obtaining of powders for an optimal diffraction experiment. Nevertheless, the information gathered about phase composition of the samples correlates with the magnetometry outcome, detailed in a further paragraph, and is vital in understanding those results.

### 3.4. DSC Results

**Figure 6:**
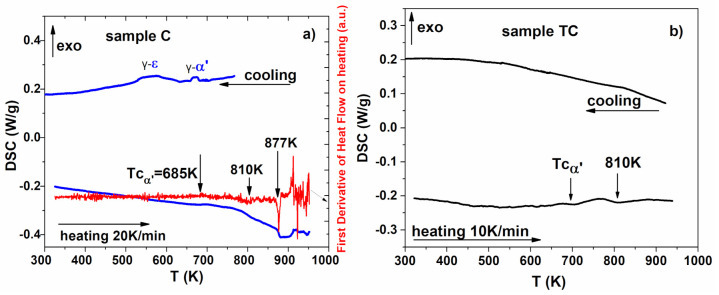
DSC scans versus temperature on heating and cooling for (**a**) sample C and (**b**) sample TC.

DSC measurements performed in a temperature range between 300 K and 950 K show the reverse behavior of MT for C and TC samples (Figure 6a, respectively, b) during the heating with 20 K/min and, respectively, with 10 K/min, in a protective He atmosphere.

On heating, the sample C show an inflexion around 685 K, attributed to the Curie temperature of the α’ phase, which may be associated with the temperature dependence of the magnetization at high temperatures (see High Temperature Thermo-Magnetic Measurements subsection). By further heating of the sample, a wide and complex endothermal peak appears which can be better explained by a correlation with the magnetization data M (T) and dM/dT (from High Temperature Thermo-Magnetic Measurements subsection). This wide peak is generated by the α’-γ transition (around 800 K) due to the thermally activated atoms diffusion. When cooling back the sample, two other transformations ε-α’and γ-ε occur, observed in the RT XRD pattern of the HC sample. The reverse transformation of the ε martensite back to austenite takes place in a wide temperature interval. In a similar study, [39] it was also found that on the heating curve double peaks attributed to the ε-γ si α’-γ transitions appear, in this order, while on cooling γ-α’ and γ-ε can be identified. The transformation α’-γ and γ-α’ is the one taking place at higher temperatures.

A different behavior is observed for the TC sample. For example, at heating, DSC measurements show a Curie transition at 685 K, and one endothermal peak at 810 K. However, MT was not detected on cooling, because there are no γ, ε, α’ phases present in the XRD results (Figure 5). The differences between the transformation reported by us and those found in literature [41] arise from the processing method.

### 3.5. Magnetic Behavior

#### 3.5.1. Low Temperature Thermo-Magnetic Measurements

**Figure 7:**
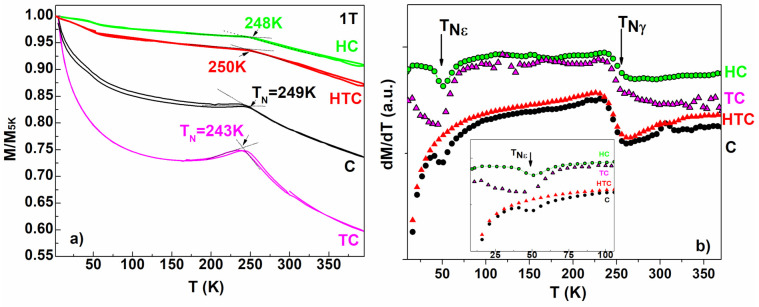
(**a**) The thermo-magnetic measurements at low temperature in 1T applied magnetic field on all investigated samples. (**b**) The first derivative of magnetization dM/dT.

Magnetic properties of Fe-Mn-Si-Cr alloys are sensitive to the Mn content [19,38,39,40], and also to the preparation route [18,19,23,24,41]. The alloys with high Mn content favour the ε-γ martensitic transformation and order antiferromagnetically below the Néel temperature [20,42,43,44,45,46]. The temperature dependence of the magnetization depicted in Figure 7a reveals a change in slope at the Néel temperature, T_N,_ from negative in paramagnetic state to positive in antiferromagnetic state and is atributed to the austenitic γ-phase [25,47,48,49,50]. The T_N_ values are rangeing between 244 K si 250 K. The derivative of the magnetization, dM/dT, exhibit another Neel temperature T_N_, aroud 50 K, which may be associated with the HSHPT induced martensite. Our results for the Neel temperature T_Nγ_ is close to the one reported in [43,44,45] for a similar Mn content, while for T_Nε_ our reported value is smaller than previously reported [44] (T_Nε_ = 173 K). At RT the magnetization did not drop to zero, and the hysterezis curves at RT (see Section 3.5.3 Hysteresis Loops subsection show that the reason for that is the presence of a ferromagnetic component, which we attribute to the phase α‘.

After a full heating-cooling circle, the measured temperature dependent magnetization shows a narrow hysteresis which is considered to depend mainly on the spin transition and interfacial effects [45]. The shape and area of this hysteresis differs from one sample to another due to different structure and morphology.

#### 3.5.2. High Temperature Thermo-Magnetic Measurements

**Figure 8:**
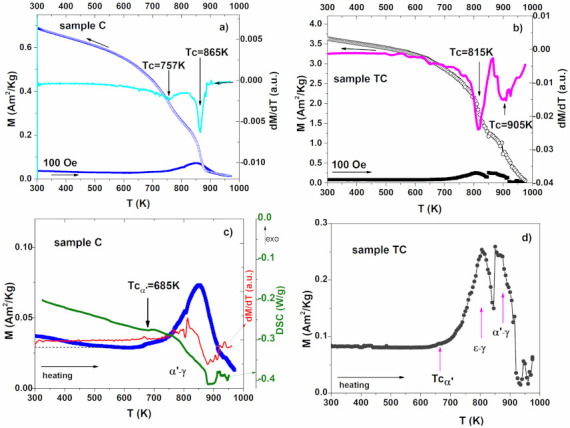
At high temperatures for (**a**) sample C and (**b**) sample TC; (**c**) Magnetization behavior superposed with DSC scan and dM/dT on the heating the sample C; (**d**) M (T) on heating of sample TC.

As already observed for the DSC measurements, the in situ investigations at high temperatures are in fact thermal treatments. Austenite reversion from ε during the heating takes place on a large temperature interval for sample C (Figure 8a,c). This phenomenon is evidenced by the slightly decrease of magnetization in M (T) curves (see Figure 8c), being obvious also in the DSC signal. The temperature increase favors the atoms diffusion and apart from the α’-γ transformation (taking place in the interval 750–800 K) and a new δ cubic phase grow, as the XRD pattern shows. The reversal martensitic transformation of TC sample indicated by temperature dependence of magnetization in Figure 8d) undergoes a two-steps process at high temperature: the ε-γ transition associated with increased magnetization at 810 K, and the α’-γ transition associated with the 880 K peak. The different behavior of the TC sample, compared with C, is due to the thermo-mechanical training process which depletes the martensite in Mn pushing the reverse transformation towards higher temperatures. Moszner et al. [46], show that the heated alloys undergo a long-range, temperature-controlled diffusion mechanism which generate zones depleted in Mn in α′-martensite regions at the end of the first stage of reversion, and a second reversion stage: the α’-γ MT, at higher temperatures. However, in the latter case, austenite reversion can be driven by both long-range and an interface-controlled mechanism. The same behavior was reported in [23,47]. At temperatures higher than 925 K, both the HTC and HC samples become paramagnetic.

At the heating towards 975 K, HTC sample also shows the segregation of a high-temperature phase above 940 K. The Curie temperature (Tc = 685 K) of the α’ phase was evaluated from the dM/dT data and is the same for both samples.

When cooled, both samples show an increase of magnetization suggesting that the new phase is ferromagnetic. From the dM/dT data one can calculate the Curie temperatures from Figure 8a,b. The thermal treatment consisted in slowly heating up the sample to 975 K (2 K/min) followed by cooling, with a short dwelling time at high temperatures. As a result, the new δ phase did not have time to grow/stabilize homogenously in the sample, which might explain the presence of two Tc temperatures that might belong to two phases with slightly different compositions. The Curie temperatures are higher for the TC sample due to the lower Mn content in the newly formed cubic phase [23,46,47].

#### 3.5.3. Hysteresis Loops

**Figure 9:**
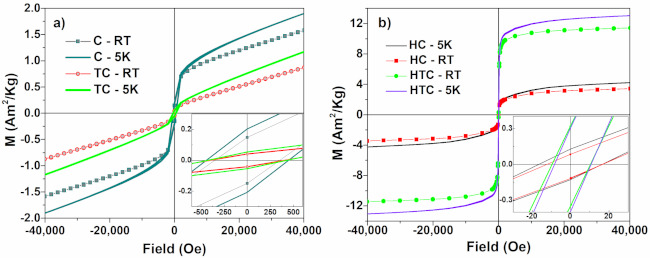
The hysteresis loops recorded at 5 K and 300 K for (**a**) C and TC samples; (**b**) the same for HC and HTC samples.

The hysteresis curves (Figure 9) for the C and TC samples, measured at 5 K and at RT, show an overlapping odd paramagnetic and ferromagnetic behavior. This is supported by the XRD results which show at RT the HSHPV induced ε martensite, the γ austenite, but also the α’ martensite.

The paramagnetic behavior is due to the γ austenite and the ε martensite (with T_N_ approx. 50 K—see Figure 7b). The ferromagnetic component can be attributed to the α’ martensite which has a high magnetic order-disorder transition temperature (680 K estimated dM/dT on heating for both samples measured at high temperatures—Figure 8c,d).

After high temperature in situ thermal treatments the saturation magnetization reaches about 3.4 emu/g for the HC samples and 11.4 emu/g for the HTC sample. Instead, the coercivity decreases from 360 Oe for the C sample to only 14 Oe for the TC sample, respectively, from 400 Oe for the HC sample to 10 Oe in HTC sample. The magnetic behavior of the HC and HTC samples become soft magnetic, due to the segregation and growth of the δ ferromagnetic cubic phase to the detriment of the ferromagnetic α’ phase, but also due to the paramagnetic γ si ε phases. The values are in agreement with [47], which reported a saturation magnetization of only 3 emu/g lower for an alloy with 17.6 wt.%Mn, and also in agreement with [48] which measured magnetic saturation values at room temperature of 11.32 emu/g for Fe-30Mn-6Si (wt.%) alloy and 18.34 emu/g for Fe- 30Mn-6Si-5Cr (wt.%) alloy.

### 3.6. Transport Properties

The transport properties of shape memory alloys depend on the electronic spectrum, electron-magnon, electron-phonon, spin-orbit couplings, polarization rotation and also on the crystal structure and phase transformations [49]. In ferromagnetic alloys SMAs the phase equilibrium is influenced by the shape of the 3d electron state density in the vicinity of the Fermi level, while the Jahn-Teller effect seems to be at the origin of the structural phase transition [50]. In this context, the transport properties of ferromagnetic shape memory alloys during the MT pose an intricate problem, as the hysteretic anomaly of the resistivity (observed also on our samples) around MT suggests [8].

**Figure 10:**
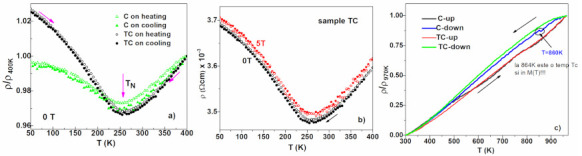
(**a**) normalized temperature dependence of the resistivity for samples C and TC; (**b**) resistivity variation with temperature in zero and 5T; (**c**) the variation of resistance with temperature at high temperature.

The low temperature dependence of resistivity for samples C and TC (shown in Figure 10) is influenced mainly by the antiferromagnetic ordering of the spins below T_N_ = 244 K, specific to the austenitic γ phase [43]. On cooling, until the antiferromagnetic order sets in, the resistivity has a metallic behavior, but at lower temperatures, an abnormal increase of the resistivity is observed. According to [51] this behavior is explained to a large extent by two factors: an effective reduction in the number of conduction electrons, originating from a truncation of the Fermi surface due to the antiferromagnetic energy gap below *T_N_*, and the increase of the magnetic scattering caused by the occurrence of a localised net moment on Fe atoms. The Neel temperature (244 K) determined from the thermo-resistivity measurements; Figure 10 is consistent with the temperature resulting from the thermomagnetic measurements (Figure 7a). Noteworthy, the transport properties of the Fe_57_Mn_27_Si_11_Cr_5_ (at %) alloy processed via HSHPT agree well with the specific behavior of Fe-Mn-Si alloys reported in literature [51,52].

The presence of a small thermal hysteresis is also remarkable. As in the case of the temperature dependence of the magnetization, it can be associated with the presence of the martensitic phase α‘.

If in other types of SMAs the resistivity decreases when the magnetic field is applied [8,53], in the FeMnSiCr alloy processed by HSHPT technique, there is an increase of the resistivity when a magnetic field is applied—Figure 10b. Thermo—resistivity measurements performed at high temperatures from Figure 10c reveal a metallic behavior for both samples: the resistivity decreases linearly on cooling due to the phonon scattering [20].

## 4. Conclusions

The effects of HSHPT and in situ thermal treatment on the properties of Fe_57_Mn_27_Si_11_Cr_5_ (at %) alloy were investigated. Microstructural investigations show that HSHPT produces a grain refinement with a wide grain size range, the appearance of needle-shaped ε-martensite (50 nm to 120 nm wide) and various defects. The crystalline structure of the HSHPT samples consists mainly of the austenite γ with ε and α’ martensite secondary phases, while the thermal treatments promote a bcc-ferrite type phase with traces of γ and ε. As an effect, the temperature dependence of magnetization shows at low temperature an anomaly specific to the antiferromagnetic transition of the austenitic γ-phase (~244 K) in all samples, which correlates to the thermo-resistivity measurements that show a metallic behavior down to T_N_ and an anomalous increase below T_N_ mainly due to the phonon scattering on spins. At room temperature, the overall behavior of magnetisation suggests the existence of two overlaying magnetic phases: the paramagnetic one (associated with austenite and ε martensite) and a magnetic ordered phase which gives a finite magnetization at RT, associated with α’ martensite. High temperature measurements act as in situ thermal treatments and lead to the formation of a δ bcc-ferrite type phase with ferromagnetic behavior, reflected by the temperature dependence of the magnetization.

## Data Availability

Data can be provided by the corresponding author upon reasonable request.
